# Ionic liquids and their bases: Striking differences in the dynamic heterogeneity near the glass transition

**DOI:** 10.1038/srep16876

**Published:** 2015-11-19

**Authors:** K. Grzybowska, A. Grzybowski, Z. Wojnarowska, J. Knapik, M. Paluch

**Affiliations:** 1 Institute of Physics, University of Silesia, ul. Uniwersytecka 4, 40-007 Katowice, Poland; 2 Silesian Center for Education and Interdisciplinary Research, ul. 75 Pułku Piechoty 1a, 41-500 Chorzów, Poland

## Abstract

Ionic liquids (ILs) constitute an active field of research due to their important applications. A challenge for these investigations is to explore properties of ILs near the glass transition temperature T_g_, which still require our better understanding. To shed a new light on the issues, we measured ILs and their base counterparts using the temperature modulated calorimetry. We performed a comparative analysis of the dynamic heterogeneity at T_g_ for bases and their salts with a simple monoatomic anion (Cl^–^). Each pair of ionic and non-ionic liquids is characterized by nearly the same chemical structure but their intermolecular interactions are completely different. We found that the size of the dynamic heterogeneity of ILs near T_g_ is considerably smaller than that established for their dipolar counterparts. Further results obtained for several other ILs near T_g_ additionally strengthen the conclusion about the relatively small size of the dynamic heterogeneity of molecular systems dominated by electrostatic interactions. Our finding opens up new perspectives on designing different material properties depending on intermolecular interaction types.

Understanding the liquid–glass transition phenomenon still remains a major challenge of the condensed matter science. If a liquid is cooling down sufficiently rapidly to omit its crystallization one can observe a dramatic increase in viscosity or structural relaxation time on approaching the glass transition. Near the glass transition temperature T_g_ the dynamics freezes drastically while the structure of the system changes only slightly in contrast to the first-order phase transition such as crystallization. The extreme slowdown in molecular dynamics is often explained by the correlated motions of the neighboring molecules which results in the appearance of cooperatively rearranging regions (CRR) introduced in Adam–Gibbs theory^1^, CRR has been defined as a group of molecules that can rearrange itself into a different configuration independently of its environment. The size of these cooperative domains increases with decreasing temperature, which denotes that larger and larger groups of molecules in a supercooled liquid are moving in a cooperative manner on reaching the glassy state. Therefore, it is often regarded that CRRs play a central role in the molecular dynamics, which becomes heterogeneous in both time and space domains near the liquid-glass transition. Although the spatially heterogeneous picture of molecular dynamics of supercooled liquids has been extensively developed since 1965 and become a paradigm in the study of physicochemical phenomena that occur near T_g_, the dynamic heterogeneity concept is still fervently debated. In the last several decades, different ways have been suggested to quantify the characteristic length scale of the spatially heterogeneous dynamics^2^. It is worth noting that direct experimental measurements of the size of the dynamic heterogeneity, mainly available by using the 4D-NMR technique, are complex and have been performed at temperatures relatively far above T_g_^3^, where the size of the dynamic heterogeneity is relatively small. Therefore, the size of the dynamic heterogeneity of real materials at the glass transition is usually evaluated by means of different estimates. A useful way to derive such estimates relies on the fluctuation-dissipation theorem, which has been exploited by both Donth^4^, and Berthier *et al.*^5^, The authors considered respectively the entropy and enthalpy fluctuations to formulate the acknowledged methods for evaluating the size of the dynamic heterogeneity or the number of dynamically correlated molecules. The approach based on entropy fluctuations, which requires data measured by using only one experimental technique, i.e., calorimetry, will be further discussed in detail.

Despite a lot of effort put into studying the dynamic heterogeneity of various model and real supercooled liquids in the last half-century^1^,^6^,^7^,^8^,^9^, the nature of the spatially heterogeneous behavior of molecular dynamics has not been completely recognized yet. One of the fundamental problems in this field, the solution of which is urgently needed, is the question of *how different kinds of intermolecular interactions affect the dynamic heterogeneity of supercooled liquids*. Until recently, many systems that belong to different material groups, such as van der Waals liquids, oxides, polymers, and hydrogen-bonded liquids, have been examined by using different methods for evaluating the dynamic heterogeneity. Based on the contributions to the dynamic heterogeneity induced by both the entropy and enthalpy fluctuations, the different authors^10^,^11^,^12^,^13^,^14^, found that the typical number of dynamically correlated molecules *N*_*α*_ near *T*_*g*_ is of the order of 10^2^ particles (considered in case of polymers usually as polymer repeating units). Depending on the material group, characterized by specific intermolecular interactions, *N*_*α*_ at *T*_*g*_ ranges approximately from 80 to 300 for van der Waals liquids, from 70 to 200 for H-bonded liquids, from 200 to 800 for polymers, and from 400 to 600 for oxides. Various attempts have been made^5^,^12^,^15^,^16^,^17^,^18^,^19^, at correlating the size of the dynamic heterogeneity with other characteristic properties of glass formers such as the fragility parameter, the activation volume, the nonexponentiality parameter of relaxation function as well as the difference between T_g_ and the dynamic crossover temperature below which the molecular dynamics is assumed to be heterogeneous. However, the study of the dynamic heterogeneity of ionic liquids, which are currently of great interest from both the application and cognitive viewpoints, has been only initiated. In a few recent years, ionic liquids have been confirmed to be structurally heterogeneous due to the existence of ionic and hydrophobic domains in the molecular systems^20^,^21^. The dynamic heterogeneity of ionic liquids at room temperature has been preliminarily suggested by using molecular dynamics simulations^22^. Very recently, Zheng *et al.*^23^ used the femtosecond IR spectroscopy to elucidate the local structural dynamics in protic alkylammonium-based ionic liquids and argued that these systems are not only structurally but also dynamically heterogeneous. Until recently, any comparative investigations have not been conducted to find how the size of the system dynamic heterogeneity changes depending on whether or not the electrostatic interactions govern the system molecular dynamics if there are no significant differences in the chemical structures of the examined systems. In this paper, we perform the comparative analysis of such selected ionic liquids and their bases to reliably check how the different kinds of intermolecular interactions in these systems influence the dynamic heterogeneity of molecular dynamics at the glass transition temperature.

## Research Idea

To study the effect of electrostatic interactions on the dynamic heterogeneity at the glass transition, we have carefully collected a unique set of glass formers, which includes several pairs of ionic liquids (hydrochloride salts) and their bases. The selected materials have also important applications because they belong to four groups of the following pharmaceuticals: (*i*) cimetidine base and cimetidine hydrochloride (inhibitors of gastric acid secretion), (*ii*) tramadol and tramadol hydrochloride (analgesic drugs)*, (iii)* carvedilol and its hydrochloride salt (cardiac drugs), and *(iv)* prilocaine and prilocaine hydrochloride (local anesthetic agents). These drugs, which initially were crystalline materials, after melting them, have been transformed to the non–ionic liquids (in case of bases) and the protic ionic liquids (in case of hydrochloride salts). All examined protic ionic liquids with the small monoatomic anion Cl^−^are formed by a proton transfer from the HCl acid to a base as follows: Base + HCl → HBase^+^ + Cl^−^. Therefore, each pair of the ionic and non-ionic pharmaceuticals is characterized by nearly the same chemical structure (i.e., the cation of any examined ionic liquids has only one excess proton in comparison to its base) but their intermolecular interactions are completely different. In the case of bases, the electrostatic interactions are negligible and molecular dynamics of these materials can be described by the Lennard-Jones kind of intermolecular potential to a good approximation. In contrast to the bases, the molecular dynamics of their ionic counterparts involves the long-range electrostatic intermolecular interactions that dominate the weak type of short-range interactions (van der Waals forces or/and hydrogen bonds). The comparative studies have been extended to other several representatives of ionic systems, including protic and aprotic ionic liquids, to gain a better insight into the dynamic heterogeneity of different materials, the molecular dynamics of which is dominated by electrostatic interactions. It should be emphasized that we have tested experimentally only materials of high purity (at least 98%), including only sufficiently well-ionized protic ionic liquids^24^, for which 14 < ∆pK_a_ < 16.5. All the ionic and non-ionic systems were purchased from commercial companies: carvedilol HCl, tramadol HCl and their bases from Polpharma (Starogard Gdański, Poland) and the others from Sigma-Aldrich.

To evaluate the difference between characteristic numbers of dynamically correlated molecules for ionic and dipolar liquids at T_g_, we apply one of the most commonly used measures of the size of the dynamic heterogeneity, which has been formulated by Donth^4^,^25^. This approach based on the fluctuation-dissipation theorem relates the entropy fluctuations to the specific heat capacity C_p_ measured within the temperature range in which the glass transition occurs. The width of the step in the temperature dependence C_p_(T) at the glass transition has been suggested as the size of temperature fluctuations^12^. Consequently, the number of dynamically correlated particles, 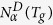, or the corresponding volume of the area occupied by the dynamically correlated molecules, 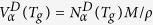, can be estimated by the following equation:


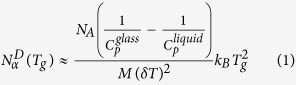


where T_g_ is the glass transition temperature, ρ–material density, k_B_–Boltzman constant, M–molar mass, 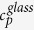, 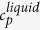
– the isobaric heat capacities of glass and liquid at T_g_, and δT is the average temperature fluctuation, which is related to the dynamic glass transition. It is worth noting that the Donth formula was originally derived^4^ in the NVT statistical ensemble and Eq. (1) is its representation in the NPT statistical ensemble^11^, which is proper to use in typical experimental conditions. Moreover, it should be mentioned that Donth^4^ originally formulated Eq. (1) to evaluate the size of CRRs assumed by the Adam-Gibbs model. However, already Hempel *et al.*^12^ have strongly related the measure defined by Eq. (1) to the size or at least a quantitative indication of the dynamic heterogeneity, broadly applying Eq. (1) to various materials from different material groups. It should be stressed that many applications of Eq. (1) showed that the Donth method yields values of 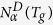, which are considerably larger than those obtained for the lower limit z^*^ to the size of a cooperative subsystem suggested by Adam and Gibbs^1^. While 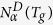 can be equal to tens or even hundreds of molecules^12^,^13^, the values of the cooperativity parameter z^*^ of the Adam-Gibbs model, which can be also evaluated by using heat capacity data, usually do not exceed 10 molecules at T_g_^26^,^27^,^28^. These discrepancies can be rationalized by considering Eq. (1) as an alternative estimate of the size of the dynamic heterogeneity to the estimate suggested by Berthier *et al.*^5^ for the measure of the dynamic heterogeneity defined by the height of the peak of the four-point dynamic susceptibility function, because both the estimates have similar theoretical grounds (i.e., the fluctuation-dissipation theorem applied respectively to the entropy and enthalpy fluctuations)^11^ and yield consistent quantitative results^29^. By studying the shapes of CRRs glass-forming liquids in terms of random first order transition theory, the Wolynes team has drawn even stronger conclusions^30^. The authors have suggested that the CRRs involve about 100 molecules near T_g_, which is considerably larger than the predictions of the CRR size via the cooperativity parameter z^*^ of the Adam-Gibbs theory, but very well corresponds to the size of the dynamic heterogeneity quantified by using the formalism of the four-point correlation function. For all the reasons, Eq. (1) is regarded in this paper as a good estimate of the number of dynamically correlated molecules near T_g_.

To determine the accurate temperature dependences of the isobaric heat capacity of examined systems near their glass transition, we exploited the stochastic temperature-modulated differential scanning calorimetry (TMDSC) technique implemented by Mettler-Toledo (TOPEM^®^). The quenched sample was heated at rate of 0.5 K/min. In the experiment, a temperature amplitude of the pulses of 0.5 K was selected with a switching time range with minimum and maximum values of 15 and 30s, respectively. We adjusted our evaluations of the temperature dependence of the quasi-static heat capacity C_p_(T) using a sapphire reference curve.

## Results and Discussion

**Figure 1:**
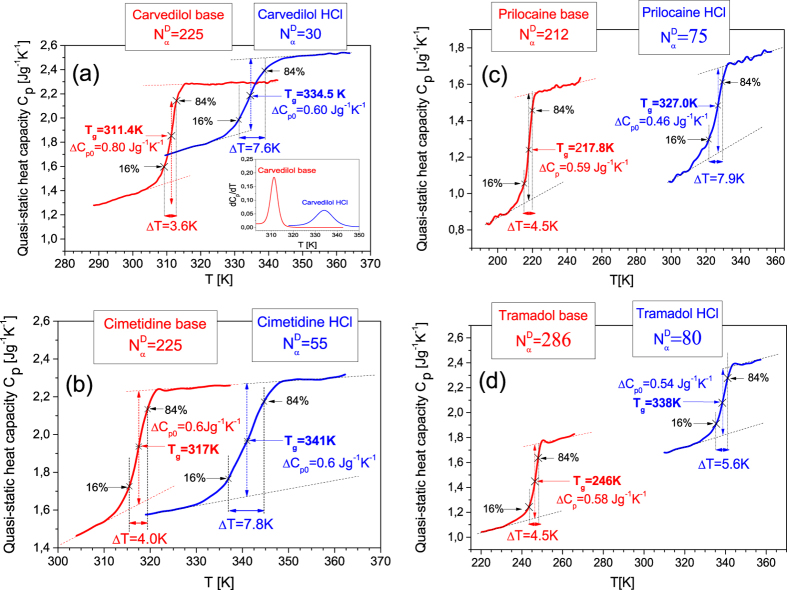
Temperature dependences of quasi-static specific heat capacity C_p_ for the examined pairs of ionic liquids (hydrochloride salts) and their base counterparts: (**a**) carvedilol, (**b**) cimetidine, (**c**) prilocaine, and (**d**) tramadol, near their glass transitions. The inset in panel (**a**) shows the temperature dependences of the derivative of C_p_ with respect to temperature for carvedilol base and carvedilol HCl.

The experimental results of the thermal analyses are presented in Fig. 1 for each investigated pair of ionic liquid and its non-ionic counterpart. Comparing the obtained dependences C_p_(T) one can make a striking observation that the widths of the glass transition are much larger for ionic liquids than those for their bases, which may strongly influence the number of dynamically correlated molecules. Differences in the shape of the dependence C_p_(T) in the glass transition region can be additionally demonstrated by plotting the temperature dependence of the derivative dC_p_(T)/dT, which reflects both the width of the glass transition and the changes in the value of C_p_ during the glass transition. As an example, such a derivative analysis for carvedilol base and carvedilol HCl is presented in the inset in Fig. 1(a), which clearly shows that the dependence dC_p_(T)/dT is broader and lower for carvedilol HCl than that for its base counterpart. The smaller maximal value of the derivative dC_p_/dT determined at T_g_ for carvedilol HCl in comparison with that for carvedilol base corresponds to the slower decrease in C_p_ during the vitrification of the liquid carvedilol HCl than that established for its base counterpart at the liquid-glass transition.

**Figure 2:**
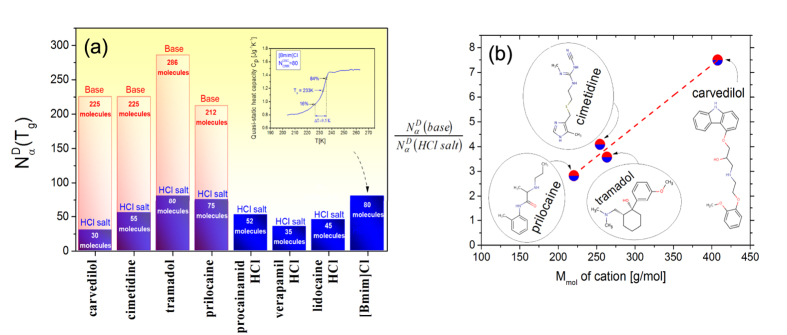
(**a**) Comparison of the numbers of correlated molecules 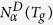 for examined pairs of dipolar bases (red bars) to their hydrochloride salts (blue bars) as well as to 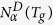 of other protic and one aprotic ionic liquids with monoatomic Cl^−^ (blue bars). The inset shows the temperature dependence of quasi-static heat capacity C_p_ for the aprotic ionic liquid [Bmim]Cl. (**b**) Correlation between the ratio of the numbers of dynamically correlated molecules for a base 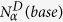 and its hydrochloride pharmaceutical salt 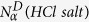 at *T*_*g*_ and the molecular weight *M*_*mol*_ of its cation.

According to the Donth model (Eq. 1), the measure of the glass transition width ∆T is the temperature interval within which the heat capacity C_p_ changes from 16 to 84% of the total heat capacity step ∆C_p_ at T_g_^12^,^31^. We found that the glass transition widths are in the separate ranges 5.6 K ≤ ∆T ≤ 7.9 K and 3.6 K ≤ ∆T ≤ 4.5 K for ionic and non-ionic liquids, respectively. Taking into account that the average temperature fluctuation employed in Eq. 1 is determined as δT = ∆T/2.5 (because we performed the DSC experiments during heating the samples), we evaluated the number of dynamically correlated molecules at T_g_, 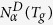, for all examined materials. As a result, we established significant differences in the size of the dynamic heterogeneity depending on the intermolecular interactions dominating in molecular dynamics of the considered systems. We found (see Fig. 2(a)) that the numbers of correlated molecules are considerably smaller for ionic liquids (30 ≤ 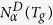 ≤ 80) than those for their base counterparts (212 ≤ 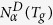 ≤ 286). It is worth noting that the small sizes of the dynamic heterogeneity of the ionic systems have been achieved despite their considerably higher glass transition temperatures in comparison with the values of T_g_ for the corresponding bases (see Fig. 1), which could result in a decrease in 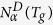 according to Eq. (1). It means that the inverse reduced average temperature fluctuation 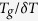, which influences 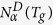 via the proportionality 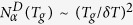, is rather dominated by the large value of the average temperature fluctuation 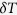
(reflected in the width of the glass transition in C_p_(T)) than by the high value of *T*_*g*_ in case of the examined ionic liquids.

The relative change in the dynamic heterogeneity of an ionic liquid and its base can be well demonstrated by the ratio 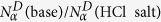, which indicates how many times the size of the dynamic heterogeneity of the ionic liquid is less than that established for its base counterpart. We found a linear correlation (see Fig. 2(b)) between the ratio of the numbers of dynamically correlated particles for a base and its hydrochloride pharmaceutical salt and the molecular weight of its cation. Although the molecular weight cannot be straightforwardly regarded as a sufficient measure of the complexity of the molecular chemical structure of cations, we can suggest that the decrease in the size of the dynamic heterogeneity of a HCl ionic liquid in comparison with that found for its dipolar counterpart depends on the molecular complexity of the salt cation. However, further investigations are required for more systems to verify a general character of the suggestion.

To give an additional evidence that the systems, the molecular dynamics of which is dominated by electrostatic interactions, are characterized by the small size of the dynamic heterogeneity, we have applied the Donth model to analyze also other ionic liquids of a similar kind (i.e., belonging to well-ionized HCl salts). Although their base counterparts have not been possessed by us or they have turned out to be poor glass formers (some of them easily recrystallize from the supercooled liquid state), the obtained results for the additionally examined ionic liquids support our findings based on the comparative analysis of 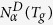 performed for the ionic and dipolar systems. For these ionic liquids, we also observe the large widths of the glass transition in C_p_(T) and the small numbers of the dynamically correlated molecules. For the additionally examined protic ionic liquids, we have established (∆T = 7.3 K, 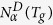 =45) for lidocaine HCl, (∆T = 6.7 K, 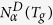 =52) for procainamid HCl, and (∆T = 5.3 K, 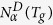 =35) for verapamil HCl. Besides 7 protic ionic liquids and 4 bases, we have measured and analyzed 2 aprotic ionic liquids such as 1-butyl-3-methylimidazolium chloride ([Bmim]Cl) and hexafluorophosphate ([Bmim][PF_6_]). The number of dynamically correlated molecules obtained for all bases and HCl salts at *T*_*g*_ have been compared in Fig. 2(a), which also demonstrates (in the inset) the large width of the glass transition (∆T = 9.5 K) accompanied with a relatively small number 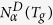 =80 for [Bmim]Cl. A preliminary test of the effect of different anions (the spherical multiatomic [PF_6_]^−^ versus the monoatomic Cl^−^) on the size of the dynamic heterogeneity yields only a small increase in 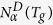 = 95 for [Bmim][PF_6_] in comparison with [Bmim]Cl, although ∆T = 4.2 K for [Bmim][PF_6_] is more than two times less than that for its chloride counterpart. Using literature data^32^, we have also analyzed 3 other aprotic ionic liquids (consisted of simple cations and anions), finding (∆T = 11 K, 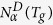 = 33) for calcium nitrate, (∆T = 11 K, 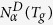 = 31) for cadmium nitrate, and (∆T = 20.3 K, 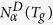 = 15) for magnesium acetate. Thus, the results of the analysis of 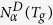 based on DSC measurements performed by us and reported by other authors show that the spatially heterogeneous molecular dynamics is subject to some kind of homogenization if the electrostatic interactions dominate the molecular dynamics.

**Figure 3:**
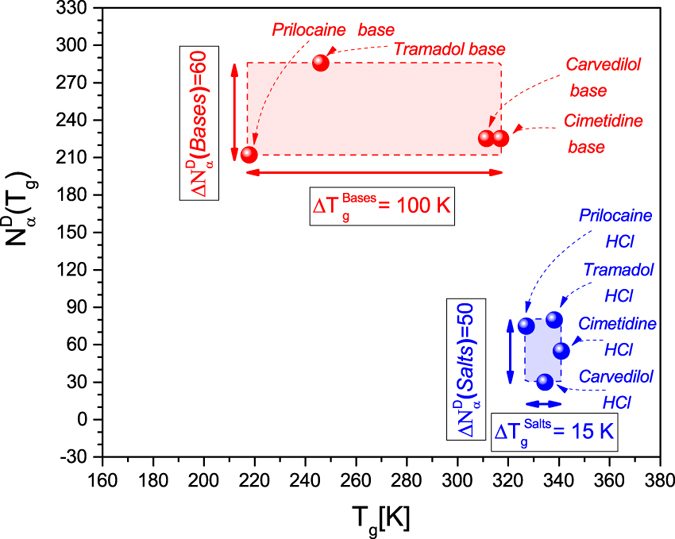
Comparison of the numbers of dynamically correlated molecules 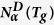 and the glass transition temperatures *T*_*g*_ for the examined pairs of the ionic liquids (blue points) and their base counterparts (red points).

Another interesting observation can be made from the comparison of 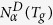 and the glass transition temperature T_g_, which have been determined for the investigated pairs of the ionic and non-ionic liquids. As can be seen in Fig. 3, besides the considerably smaller 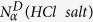 than 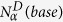, the domination of the electrostatic interactions in molecular dynamics of the examined ionic liquids results in a small diversity of the glass transition temperatures for the HCl salts (∆T_g_ = 15 K) in comparison with those established for their base counterparts, which vary within the range of ∆T_g_ = 100 K. This effect is combined with the similar and relatively small diversities in the numbers of the dynamically correlated molecules for both groups of the examined materials, i.e., 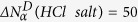 and 
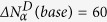
. However, the dynamic length scale of the molecular dynamics (followed from the correlation volume 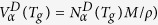 evaluated at the same time scale of molecular dynamics (defined by the same structural relaxation time related to the glass transition) strongly depends on the kind of intermolecular interactions, because 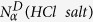 considerably differs from 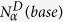 for each tested pair of base and its HCl salt.

In recent years, a great interest has been attracted by a possible relationship between structural and dynamic heterogeneities in ILs^20^,^21^,^22^,^23^,^33^,^34^,^35^,^36^,^37^. To consider the crucial issue for better understanding the molecular dynamics of ILs, we have evaluated the dynamic length scales via Eq. (1), taking carvedilol base and its HCl salt as examples and making an assumption that the correlation volume occupied by 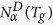 molecules is the volume of a cube, 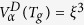. To determine the correlation volume 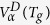, we have exploited experimental PVT data^38^,^39^, parametrized for carvedilol base and its HCl salt by an equation of state recently derived^40^ for supercooled liquids. The needed values of density for this pair of non-ionic and ionic liquids at *T*_*g*_ at ambient pressure have been calculated from Eq. (9) in ref. 40 with the values of its parameters earlier reported for carvedilol base^19^ and found herein for carvedilol HCl as follows, *A*_0_ = (0.8005 ± 0.0001) cm^3^/g, *A*_*1*_ = (4.45 ± 0.07) · 10^−4^ cm^3^/(g · K), *A*_*2*_ = (−7.12 ± 0.99). 10^−7^ cm^3^/(g · K^2^), 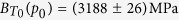, *b*_*2*_ = (2.31 ± 0.18) · 10^−3^ K^−1^, and *γ*_EOS_ = 11.92 ± 0.23, assuming that *T*_0_ = 334.5 K and *p*_0_ = 0.1 MPa in the reference state. As a result, we have established that the dynamic length scale ξ = 5.0 nm for carvedilol base is almost two times larger than that for its HCl salt at *T*_*g*_. The obtained value ξ = 2.6 nm for carvedilol HCl is identical to that found by Hempel *et al.*^12^ for the prototypical ionic liquid CKN measured by TMDSC, but it is larger than the sizes of structurally heterogeneous domains reported for ILs^21^,^33^,^41^,^42^,^43^,^44^, which do not exceed 2.0 nm. This finding well corresponds to recent theoretical and simulation studies, showing that the static (structural) length scale can be considerably less than the dynamic length scale in model systems near the glass transition^45^,^46^,^47^,^48^,^49^, which have been already confirmed experimentally in a two-dimensional colloidal glass-forming liquid^50^. Nevertheless, although the dynamic heterogeneity of ILs also seems to be spatially not limited to the structurally heterogeneous domains, we do not know whether or not the structural heterogeneities in ILs essentially affect the dynamic ones or *vice versa*.

## Conclusions

We established that the numbers of dynamically correlated molecules for protic ionic liquids with the simple monoatomic anion (Cl^–^) are considerably smaller than those for their base counterparts at T_g_. The small size of the dynamic heterogeneity of the ionic systems dominated by the long-range electrostatic interactions is reflected in the large width of the glass transition in C_p_(T), which is related to high average temperature fluctuations. Such electrostatic interactions induce the small length scale of spatially heterogeneous dynamics and the small diversity of T_g_ in the protic ionic systems. In contrast, the molecular dynamics of their base counterparts is dominated by the dipole-dipole interactions, which induce the large size of the dynamic heterogeneity and the large diversity of T_g_ for these non-ionic liquids. Moreover, we found that the relative change in the dynamic heterogeneity of an ionic liquid and its base depends on the molecular complexity of the salt cation. Although the small size of the dynamic heterogeneity in molecular systems dominated by electrostatic interactions has been additionally confirmed by analyzing several other protic and aprotic ILs near T_g_, one cannot exclude such topological constraints caused by more complex anions or cations, which may result in a larger dynamic length scale of some ILs. Thus, further investigations are required to draw a general conclusion about the size of the dynamic heterogeneity in ionic liquids. Similarly, the potential interplay between structural and dynamic heterogeneities in ILs still remains a research challenge.

It is worth noting that our results presented herein and recently reported by us^51^,^52^,^53^, for the pressure effect on the dynamic heterogeneity show that the decrease in intermolecular distances due to the system compression as well as the dominating role of the electrostatic interactions in molecular dynamics of the examined protic and aprotic ionic liquids induce the decrease in the dynamic length scale of spatially heterogeneous dynamics near the glass transition. This finding can be important to the novel solutions in the material engineering, which may be developed in the future as a result of the fundamental investigations of the dynamic heterogeneity intensively conducted in the last decades.

## Additional Information

**How to cite this article**: Grzybowska, K. *et al.* Ionic liquids and their bases: Striking differences in the dynamic heterogeneity near the glass transition. *Sci. Rep.* **5**, 16876; doi: 10.1038/srep16876 (2015).
